# 

**Published:** 1800-10

**Authors:** 


### To CORRESPONDENTS.

*The Communications which have been received, shall be noticed as soon as possible.*

*The Editors will thank Mr. Samuel Thomas for his address.*

*Several of our Correspondents have favoured us with Reviews of Medical Works, and for which we should have been much obliged to them, if we professed to criticise books; but it will be obvious, that Editors, who give their names to the Public, cannot enter the lists of criticism. It is the design of our Retrospect of Books, to put it in the power of our Readers to judge for themselves; and therefore we endeavour to give the Authors of Medical Works an opportunity of being heard in their own cause, by selections from the works themselves.*

